# Alterations in protein expression and site-specific *N*-glycosylation of prostate cancer tissues

**DOI:** 10.1038/s41598-021-95417-5

**Published:** 2021-08-05

**Authors:** Simon Sugár, Gábor Tóth, Fanni Bugyi, Károly Vékey, Katalin Karászi, László Drahos, Lilla Turiák

**Affiliations:** 1 MS Proteomics Research Group, Research Centre for Natural Sciences, Eötvös Loránd Research Network, Magyar tudósok körútja 2, 1117 Budapest, Hungary; 2 Semmelweis University, Ph.D. School of Pharmaceutical Sciences, Üllői út 26, 1085 Budapest, Hungary; 3 Faculty of Chemical Technology and Biotechnology, Budapest University of Technology and Economics, Műegyetem rakpart 3, 1111 Budapest, Hungary; 4 Eötvös Loránd University, Hevesy György Ph.D. School of Chemistry, Pázmány Péter sétány 1/A, 1117 Budapest, Hungary; 5 1St Department of Pathology and Experimental Cancer Research, Semmelweis University, Üllői út 26, 1085 Budapest, Hungary

**Keywords:** Glycomics, Proteomics, Tumour biomarkers, Prostate cancer

## Abstract

Identifying molecular alterations occurring during cancer progression is essential for a deeper understanding of the underlying biological processes. Here we have analyzed cancerous and healthy prostate biopsies using nanoLC-MS(MS) to detect proteins with altered expression and *N*-glycosylation. We have identified 75 proteins with significantly changing expression during disease progression. The biological processes involved were assigned based on protein–protein interaction networks. These include cellular component organization, metabolic and localization processes. Multiple glycoproteins were identified with aberrant glycosylation in prostate cancer, where differences in glycosite-specific sialylation, fucosylation, and galactosylation were the most substantial. Many of the glycoproteins with altered *N*-glycosylation were extracellular matrix constituents, and are heavily involved in the establishment of the tumor microenvironment.

## Introduction

Prostate cancer (PCa) is the most prevalent type of cancer and the second leading cause of cancer-related deaths among males. The probability of developing PCa increases with age, the lifetime risk is 1 in 9^1^. PCa screening is based on the Prostate-Specific Antigen (PSA) test, which measures the level of PSA in blood. In the case of elevated PSA levels, further diagnosis and prognosis are determined by the histological examination of prostate tissue biopsies^2^. PSA screening, however, has relatively low specificity for PCa, which can often result in overdiagnosis and overtreatment^3^. Histology also has various limitations such as the subjective manner of the classification of the tissue sample^4^ and the inherent sampling error due to tumor heterogeneity^5^. There have been significant efforts to improve existing methods for PCa diagnosis. This includes the discovery of novel biomarkers to replace the PSA blood test^6,7^, the use of image-guided targeted biopsies^8,9^, digital pathology using Artificial Intelligence along with Machine Learning^10,11^, and precision oncology using Liquid Biopsies^12^. Tissue samples are often used in biomarker research as the starting point since they have the advantage to identify molecular alterations occurring at the site of origin of the disease.

Tumor grading is the means of classification of tissue samples based on cellular appearances. Lower grades resemble normal tissue more, while higher grades deviate to a greater extent due to lack of differentiation. For PCa, the Gleason grading system is most frequently used, which does not focus on cytological, but rather architectural patterns and considers both the least and second least differentiated patterns observed^13–15^. Pathological Grades (G) range from 1–3 while Gleason Grades (GG) range from 1 to 5. Both can be grouped into low (G1 and GG1), intermediate (G2 and GG2-3) and high-risk groups (G3 and GG4-5).

Molecular characteristics of PCa are characterized by high complexity and heterogeneity^16^. In recent years, many promising protein biomarkers have been reported^17–19^, but none of them has been implemented into clinical practice to complement or replace PSA screening, although several are currently in clinical development^20,21^. Besides changes in protein abundances of specific proteins, changes in the glycosylation of PCa glycoproteins have also been reported to be of potential diagnostic and prognostic value^22–24^.

Mass spectrometry (MS) based proteomics methodologies are reliable and widely used tools for the analysis of prostate tissues and cell lines^25–28^. The MS-based characterization of site-specific protein glycosylation however remains a challenging task both from an analytical and data analysis standpoint^29^. This is partly the reason that protein glycosylation is still a largely untapped source of cancer biomarkers^22^.

We have recently performed a comparative pilot proteomics study on PCa tissue microarrays (TMAs) to discriminate between healthy and cancerous tissues^30^. In the present study, our objective was to identify molecular changes in PCa, analyzing a large number (95) of TMA core biopsy samples. This allows us to detect relatively small differences in protein abundances with a high statistical power. We have compared protein expression levels and changes in *N*-glycosylation features among various pathological grades of PCa and healthy tissues.

## Results

**Figure 1:**
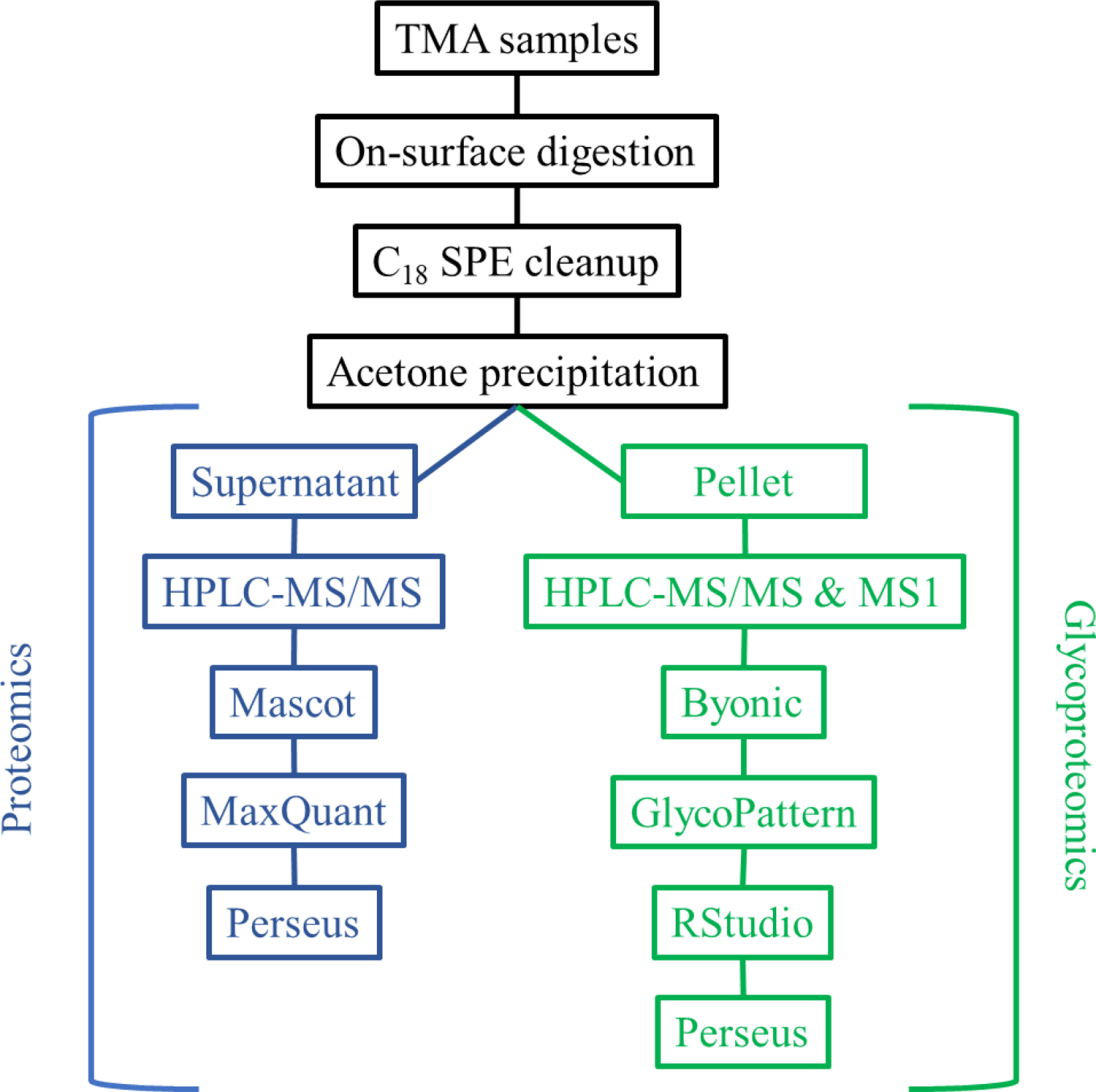
Workflow of the analysis of the TMA samples.

Protein expression levels and site-specific *N*-glycosylation of 95 tissue microarray (TMA) biopsy samples were analyzed, among which there were 9 G1, 16 G2, 24 G3, and 46 normal tissues. Digital images of a stained sample from each group are shown as examples in Supplementary Figs. S1–S4. In the case of cancerous samples both the original and an annotated image indicating cancerous and non-cancerous tissue areas are shown. The sample preparation consisted of on-surface tryptic digestion^31^ followed by C_18_ SPE cleanup and acetone precipitation for glycopeptide enrichment. After precipitation, the glycopeptide-enriched pellet fraction and the supernatant fraction containing non-glycosylated peptides were analyzed separately^32^. The workflow is summarized in Fig. 1, detailed information on each step is discussed in the “Methods” section.

The “Results” section is divided into three major parts: (i) the molecular differences between healthy and cancerous prostate tissue; (ii) the molecular changes with PCa grade progression, and differences between distinct grades and healthy tissue; (iii) and the biological processes altered in PCa. While the first two sections are based on data from both the proteomics (containing protein intensities) and glycoproteomics datasets (containing glycopeptide intensities and metrics calculated from them), the third one is based on proteomics data only. Before describing the results of the three aforementioned sections, a general characterization of the two datasets (proteomics and glycoproteomics) is provided.

MaxQuant quantified 653 proteins altogether in the 95 supernatant samples analyzed. From these, proteins that were found in less than 60% of any of the sample groups were excluded. Missing values were then imputed as described in the “Methods” section.

*N*-linked glycopeptides were quantified by GlycoPattern^33^ software using glycan and glycosite libraries constructed following Byonic searches^32^. Results were then filtered as detailed in the “Methods” section. Altogether 145 glycopeptides were quantified in 95 samples with high confidence, corresponding to 22 glycoproteins with 29 glycosites and 53 different glycans.

Protein glycosylation can be characterized by listing all the identified glycopeptides, but usually, multiple metrics are used instead^22,34^. Here we use sialylation, fucosylation, galactosylation, branching, and glycan type ratio. These simplify data interpretation and carry important biological information as well, as they are connected to various steps of *N*-glycan biosynthesis. The different metrics used in this paper are explained and summarized in Supplementary Table S1.

Over 75% of the identified glycopeptides carried complex-type glycans. More than half of these structures were biantennary, while tri- and tetra-antennary types and unmatured structures were also present. The average antenna sialylation was 20.1% across all samples, while 28.7% of antenna containing structures held at least one sialic acid. The average fucosylation was 37.8% across all samples. All 29 glycosites identified carried several different glycans, and also showed considerable diversity regarding glycan type, branching, galactosylation, fucosylation, and sialylation. To reveal changes specific to the distinct glycosites, metrics were calculated for them individually as well.

### Differences between healthy and cancerous tissues

**Figure 2:**
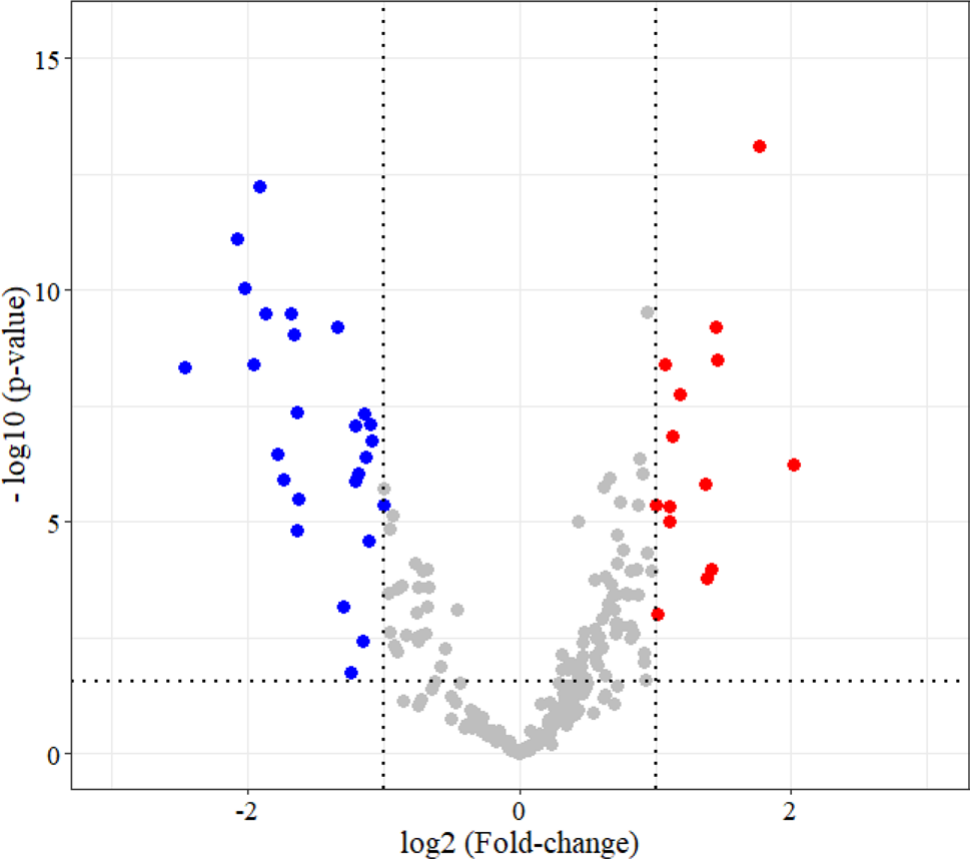
Volcano plot displaying proteins differentially expressed (fold-change at least 2) between healthy and PCa tissues. Red dots represent proteins overexpressed, while blue dots represent proteins underexpressed in PCa.

To investigate differences between healthy (normal) and cancerous (PCa) tissues, Student’s t-test was performed on proteomics and glycoproteomics data separately using 0.05 false discovery rate (FDR). Between the normal and PCa groups, 123 proteins were found to be differentially expressed, this included 72 proteins overexpressed and 51 proteins underexpressed in PCa (Supplementary Table S2). Among these, 14 showed a fold-change over 2, while 27 displayed a fold-change under 0.5 (Fig. 2).

In the glycoproteomics dataset, 7 glycopeptides were found with significantly different abundances between the normal and PCa groups (Supplementary Fig. S5), each carrying biantennary, fucosylated complex-type glycans with different levels of galactosylation and sialylation. In five cases, glycopeptide expression was lower in PCa tissues: four glycoforms of Immunoglobulin gamma-1 heavy chain (IGG1) N299, and one glycoform of Prothrombin (THRB) N121. The other two showed higher expression levels in PCa: one glycoform of Microfibril-associated glycoprotein 4 (MFAP4) N137 and one glycoform of Biglycan (PGS1) N270.

**Figure 3:**
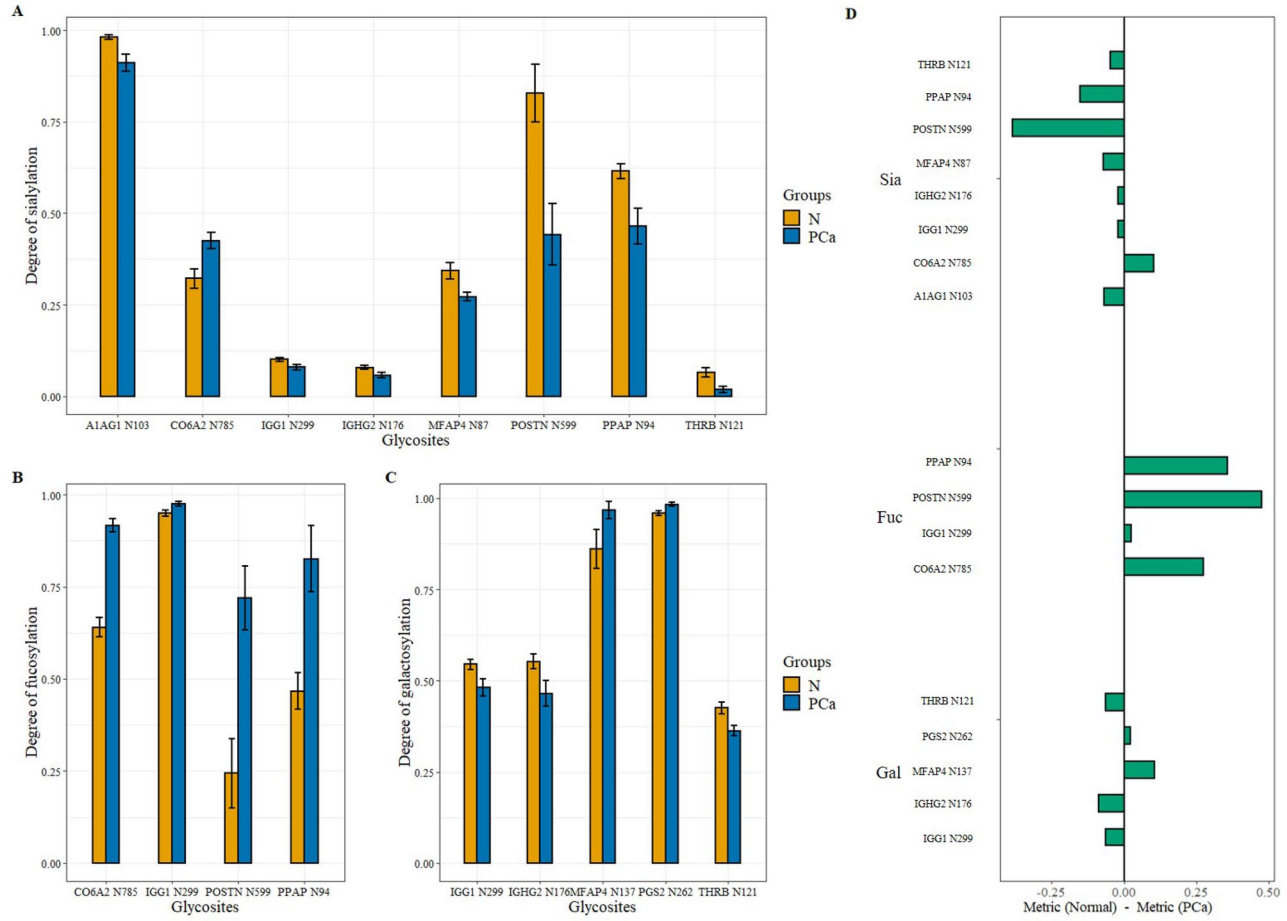
Glycosite-specific alterations in sialylation (**A**), fucosylation (**B**), and galactosylation (**C**) between healthy and PCa tissues (with standard error displayed). (**D**) summarizes the direction and volume of the differences in the case of all three metrics (Normal—PCa).

Significant differences were also detected between normal and PCa tissues when comparing the levels of sialylation, fucosylation, and galactosylation at distinct glycosites. The differences in glycosite-specific sialylation, fucosylation, and galactosylation are summarized in Fig. 3.

All but one of the eight differentially sialylated glycosites were underexpressed in PCa (Fig. 3A). The differences in sialylation were below 10% for most glycosites, except for Periostin (POSTN) N599 and Prostatic acid phosphatase (PPAP) N94 with a 38.6% and 15.1% decrease respectively, and CO6A2 N785 with a 10.3% increase in sialylation. Although only a 4.6% difference, THRB N121 showed the greatest relative change with a degree of sialylation almost 3.5 times lower in PCa than in normal tissues. Opposed to this, all four differentially fucosylated glycosites were overexpressed in PCa with the biggest differences on N785 of collagen alpha-2(VI) chain (CO6A2), POSTN N599, and PPAP N94 with a 27.7%, 47.6%, and 35.9% increase in fucosylation, respectively (Fig. 3B). The significant differences in galactosylation levels found on five glycosites (Fig. 3C) were much smaller than changes in fucosylation or sialylation, the two major ones being the increase of galactosylation at MFAP4 N137 by 10.6% and the decrease of galactosylation of Immunoglobulin heavy constant gamma 2 (IGHG2) N176 by 8.7% in case of cancerous samples. Interestingly, while changes in fucosylation always increased in the case of PCa samples (Fig. 3D), in the case of sialylation and galactosylation they did not.

### Differences among various grades of PCa and Normal tissue

To uncover molecular alterations among pathological grades and normal tissue, Analysis of Variance (ANOVA) was performed (FDR controlled at 0.05) on both proteomics and glycoproteomics data separately. For exact parameters see the “Methods” section.

**Figure 4:**
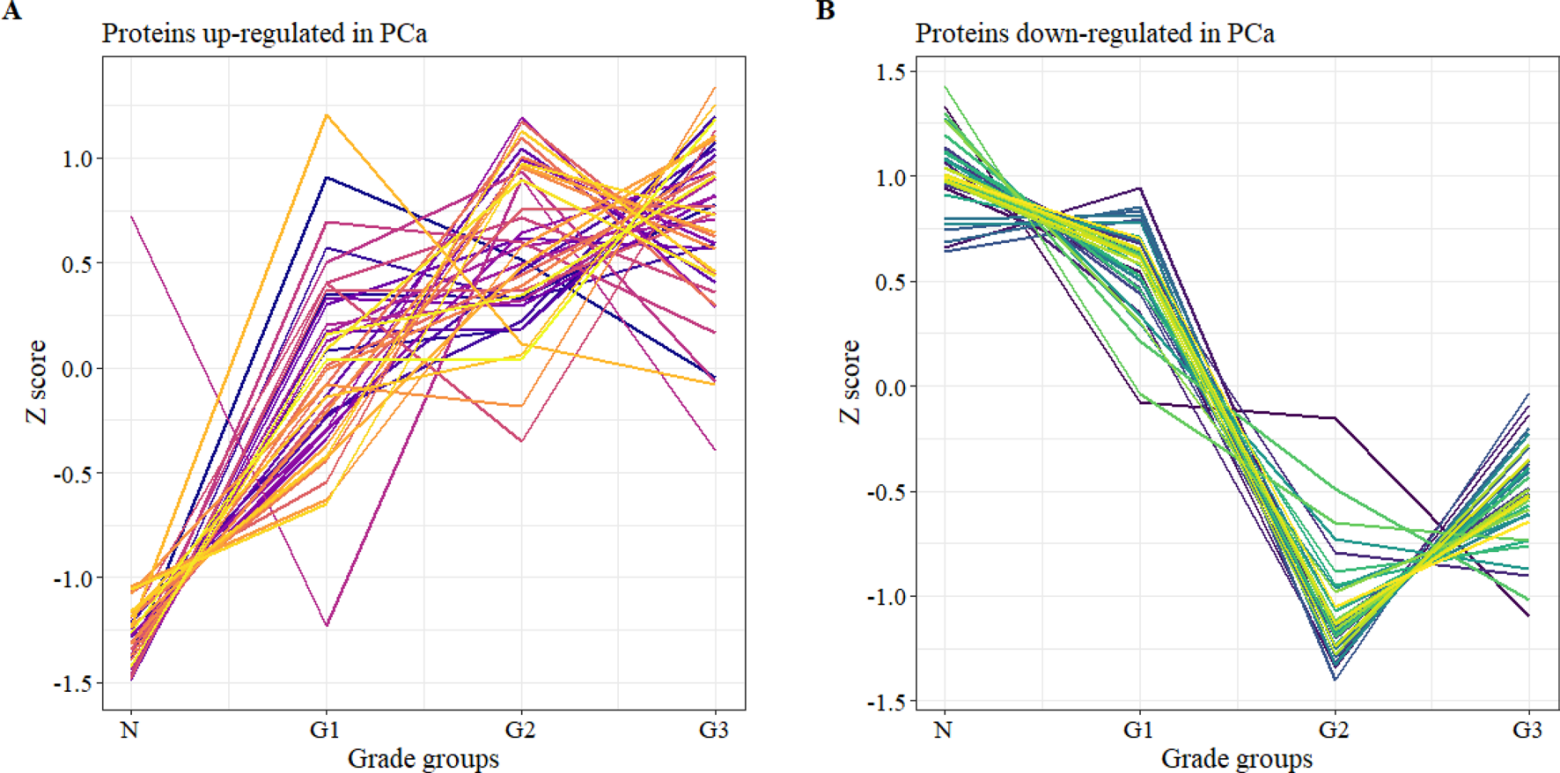
Significantly changing proteins among different grades of PCa and healthy tissue divided into two sub-groups based on hierarchical clustering: upregulated (**A**) and downregulated (**B**).

In the proteomics dataset, 75 proteins were identified with significant changes (Supplementary Table S3) among the various PCa grades and healthy tissue. Hierarchical clustering in Perseus with Spearman’s correlation revealed two distinct groups among these proteins: in 40 cases the proteins were upregulated (Fig. 4A), while in 35 cases they were downregulated (Fig. 4B) in cancer.

Afterward, a post-hoc test was performed on the 75 ANOVA significant proteins (Tukey’s Honest Significant Difference test). This revealed that most of the proteins were differentially expressed between the normal and the two high-grade groups (G2 & G3), while there were only 3 such proteins between G2 and G3, 8 proteins between G1 and G3 and 14 between normal and G1 groups. The list of these proteins is included in Supplementary Table S3 broken down into six groups corresponding to all group-wise comparison combinations. Furthermore, many of them (more than 85%) showed differential expression in not only one but multiple group comparisons (Supplementary Fig. S6).

**Figure 5:**
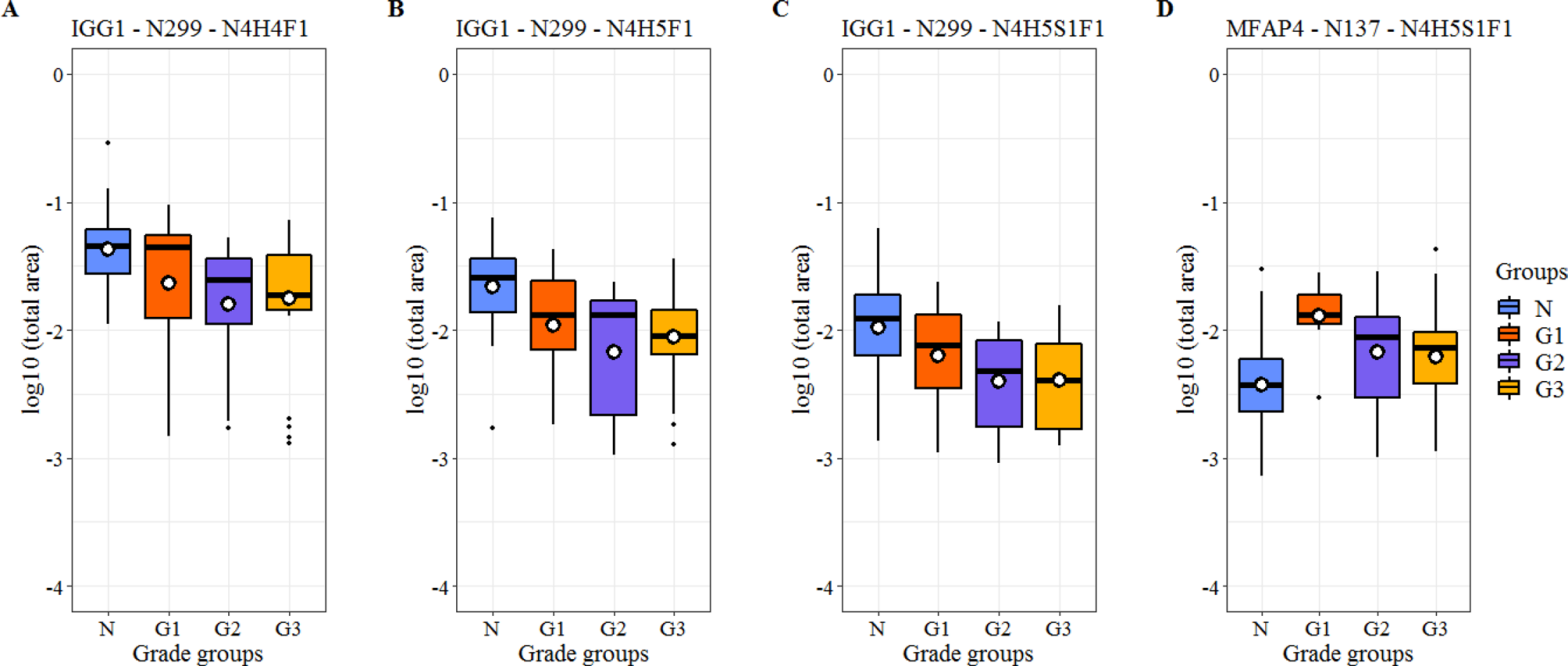
Glycopeptides with significant changes between different Grades of PCa and healthy tissues. Glycopeptides are annotated as follows: glycoprotein—glycosite—attached glycan (*H* hexose, *N* N-acetyl hexosamine, *F* fucose, *S* sialic acid units).

In the glycoproteomics dataset, ANOVA and the following post-hoc test (Tukey’s HSD) revealed 4 glycopeptides with significantly different abundances among different grades and healthy tissue. Three of them correspond to the same glycosite N299 of IGG1 and carry biantennary complex-type glycans. In all three cases, the significant differences were between the Normal—Grade 2 and Normal—Grade 3 groups, and the observed trends were similar (average correlation coefficient of 0.980). The overall amount of IGG1 glycopeptides did not change significantly with PCa progression. The fourth glycopeptide corresponds to glycosite N137 of MFAP4 and also carries a biantennary complex-type glycan. In this case, the significant difference is between the Normal—Grade 1 groups (Fig. 5).

**Figure 6:**
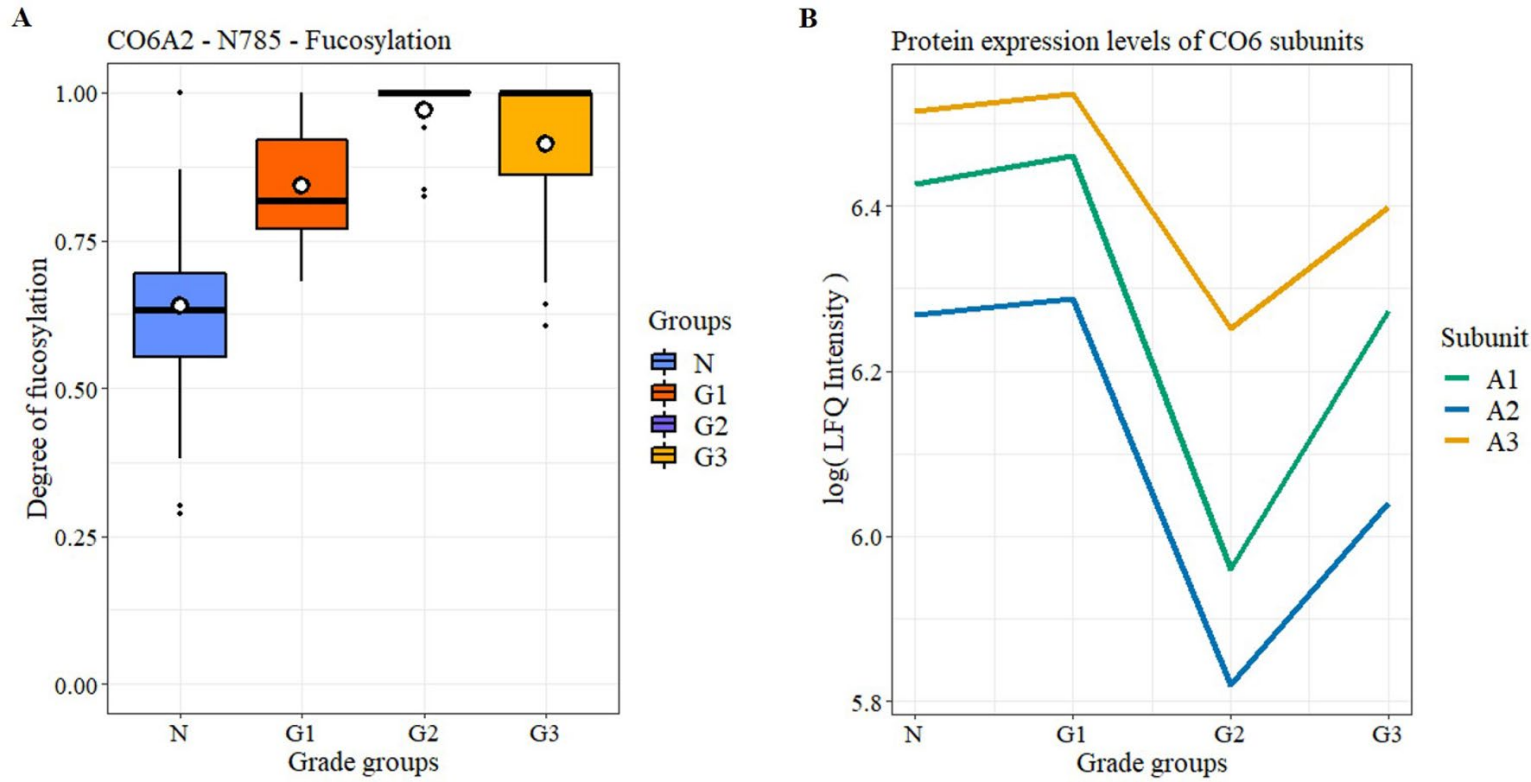
Changes in the fucosylation of CO6A2 (**A**) and in the protein expression of different CO6 subunits (**B**) between different Grades of PCa and healthy tissues.

Furthermore, regarding glycosites, ANOVA identified that the degree of fucosylation on CO6A2 N785 was different between the three Grade groups and Normal tissue. Interestingly, fucosylation shows a monotonic increase until G2 then decreases in G3 (Fig. 6A). This tendency is opposite to the changes in protein expression levels of the 3 identified CO6 subunits A1, A2, and A3 (Fig. 6B) apart from both being nearly constant between the Normal and G1 groups.

In addition to pathological grades, alterations between Gleason grades and healthy tissue were investigated as well. The number of samples analyzed in the different GG groups was as follows: 7 in GG2, 12 in GG3, 15 in GG4, and 15 in GG5. The data analysis was carried out similarly to that of pathological grades.

**Figure 7:**
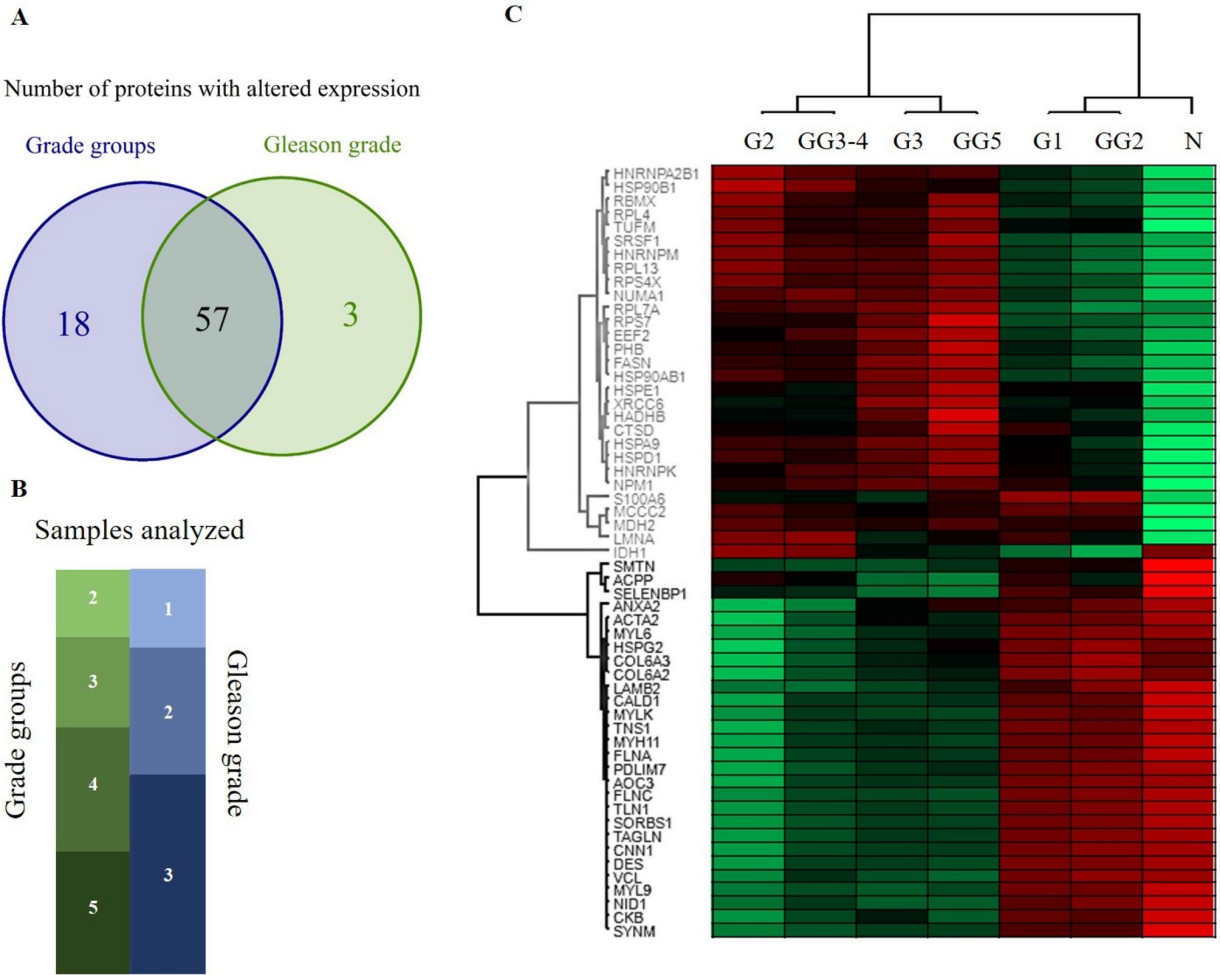
Comparison of the proteomics results based on Grade groups and Gleason grades. (**A**) Venn diagram of proteins identified as significant. **(B**) Classification of the 49 PCa samples analyzed. The size of the boxes is proportional to the sample sizes (green—Gleason grades, blue—Grade groups). (**C**) Heatmap of the 57 common proteins in both proteomics datasets. Grade groups (G1, G2, G3) and groups created from Gleason grades (GG2; GG3 and GG4; GG5).

The results of the analysis based on GG groups showed great similarity to those based on pathological grades. In the glycoproteomics dataset, the same glycosylated features were identified with significant changes, regarding both glycopeptides and glycosites. In the proteomics dataset, 60 proteins were identified as differentially expressed, opposed to 75 in the analysis based on pathological grades, with 57 common ones between the two. The overlap between these two sets of proteins and the group classifications of the 49 PCa samples are summarized in Fig. 7A,B, respectively. The correlation was also calculated for the 57 common proteins for the two datasets. Gleason grades were grouped based on the amount of overlap with pathological grades (Fig. 7B) in the following manner: GG2; GG3 and GG4; and GG5. The correlation coefficients between the GG2 – G1; GG3 and GG4 – G2; GG5 – G3 grades were 0.997, 0.970, and 0.990, respectively. The high correlation of these groups is visualized in Fig. 7C in the form of a heatmap, containing the 57 common proteins and the compared groups (clustering is based on Pearson correlation, protein intensities are depicted as Z-scores).

### Identification of altered biological processes based on proteomics

Following the identification of proteins with statistically significant changes, functional enrichment analysis was performed in STRING for GO and KEGG terms, separately for the proteins up- and downregulated in PCa (for the STRING networks see Supplementary Figs. S7 and S8). The most important terms from the resulting Protein–Protein Interaction (PPI) Networks were identified based on the Number of Genes, Strength, and FDR values, and are summarized in Table 1. The complete lists are presented in Supplementary Tables S4–S7.

**Table 1:** The most important terms in the PPI Networks from the STRING analysis.

Database	Description	Number of genes	Strength	FDR
**Protein–protein interaction networks for the 51 proteins underexpressed in PCa**				
KEGG	Focal adhesion	12	1.39	6.62E−12
KEGG	ECM-receptor interaction	6	1.47	2.49E−06
KEGG	Vascular smooth muscle contraction	6	1.3	1.44E−05
GO BP	Muscle contraction	13	1.33	6.53E−11
GO BP	Actin filament-based process	14	1.05	7.47E−09
GO BP	Cell junction assembly	9	1.43	2.94E−08
GO BP	Cellular component organization	34	0.42	1.30E−07
GO BP	Actin cytoskeleton organization	12	1.06	1.30E−07
GO BP	Extracellular matrix organization	10	1.13	6.01E−07
GO BP	Cytoskeleton organization	14	0.77	7.79E−06
GO BP	Supramolecular fiber organization	9	0.97	4.79E−05
GO BP	Platelet degranulation	6	1.27	8.66E−05
GO BP	Cell adhesion	12	0.75	8.66E−05
GO BP	Regulated exocytosis	11	0.8	8.66E−05
**Protein–protein interaction networks for the 72 proteins overexpressed in PCa**				
KEGG	Ribosome	8	1.22	4.25E−06
KEGG	Spliceosome	8	1.22	4.25E−06
GO BP	mRNA metabolic process	23	0.97	4.28E−13
GO BP	RNA splicing, via transesterification reactions	15	1.15	2.64E−10
GO BP	Protein localization to endoplasmic reticulum	9	1.3	1.76E−07
GO BP	Metabolic process	60	0.23	1.92E−07
GO BP	SRP-dependent cotranslational protein targeting to membrane	8	1.37	3.02E−07
GO BP	Translational initiation	9	1.24	3.57E−07
GO BP	Protein localization	25	0.54	8.45E−07
GO BP	Negative regulation of gene expression	23	0.57	8.87E−07
GO BP	Regulation of gene expression	38	0.36	2.04E−06
GO BP	Cellular response to cytokine stimulus	17	0.69	2.08E−06
GO BP	Localization	41	0.33	2.51E−06
GO BP	Translation	11	0.92	3.63E−06

Most of the underexpressed proteins were associated with cellular component organization (34 out of 51), while the overexpressed proteins were predominantly affiliated with metabolic processes (60 out of 72).

## Discussion

As the focus of this paper is on finding potential biomarkers through exploring alterations in the glycosylation between healthy and PCa tissues combined with proteomics data, only glycoproteins displaying significant changes are discussed individually. For these, the differences in protein expression and glycosylation are both reported, and they are compared to relevant previous studies on PCa or cancer in general. Furthermore, the most significant biological processes are also discussed.

The PPI network analysis provides information about biological processes, which are altered in PCa. The underexpressed proteins were mostly associated with cellular component organization (34 out of 51 proteins) and various processes connected to adhesion e.g.: the KEGG term “Focal adhesion” and the GO term “cell adhesion”, and muscle contraction e.g.: the KEGG term “Vascular smooth muscle contraction” and the GO term “muscle contraction”. Focal adhesion has been confirmed to be heavily involved in cancer progression^35^, while smooth muscle cells have been reported to be involved in PCa and BPH^36^. The overexpressed proteins, on the other hand, were primarily associated with metabolic processes (60 out of 72 proteins) with the GO terms “localization” and “regulation of gene expression” involving the most proteins. While altered localization of macromolecules in a cell (e.g. proteins^37^) can reportedly drive tumor development and metastasis, aberrant gene expression is known to be the principal cause of cancer^38^.

All glycoproteins with significant glycosylation changes were quantified in the proteomics part of the study by MaxQuant, but not all of them showed differential expression between Normal and PCa tissues. This suggests that altered glycosylation does not necessarily indicate glycoprotein-wise differential expression. Furthermore, neither of the metrics used for the characterization of glycosylation (listed in Supplementary Table S1) showed significant overall changes between PCa and healthy tissues. Regarding cellular localization, all the glycoproteins with significant glycosylation changes were primarily of extracellular origin, most of them were associated with the Extracellular Matrix (ECM) and consequently, the Tumor Microenvironment (TME), which is known to heavily influence cancer initiation, progression, and invasion^39^.

There are several changes in glycosylation that are known to widely occur in cancer. These include increased and altered sialylation, increased branched-glycan structures, and fucosylation^40,41^. Also, there have been many PCa glycome-specific changes reported before^42^, e.g.: the expression of oligomannosidic glycans in the tumor region in late-stage PCa^43^. These changes however reflect only overall tendencies, they are not necessarily true for all of the glycosylation sites, as our results clearly demonstrate.

In previous studies, serum sialylation has been linked to pathological grade and elevated sialic acid levels to bone metastasis^44^. In tissues, however, overall sialylation levels have been reported to be constant across different grades of cancer^22^. Our results suggest the same, the average sialylation levels were very similar throughout the different sample groups, but there were significant differences detected on several glycosylation sites. Most of them showed a decrease in sialylation except for CO6A2 N785, which showed an overall increase and significant differences between the different pathological grade groups. Also, proteomics results revealed that CO6A1, CO6A2, and CO6A3 expression levels significantly changed with PCa progression in a similar manner. This is highlighted by the fact, that CO6A1 has been reported to have an important role in tumor growth, and the molecular etiology of Castration-Resistant Prostate Cancer^45^.

Apart from serum, PCa cell lines have also been used before to identify diagnostic markers, and site-specific changes in fucosylation have been reported in PC3 and LNCap cell lines^46^. This aligns with our findings, as we have also found that fucosylation increased in PCa on multiple glycosites. Also, PPAP has been demonstrated to have a significant effect on PCa cell growth^47^, and it has been hypothesized to have higher site-specific fucosylation levels in PCa patients^46^. This is supported by our data: the average fucosylation level of PPAP N94 increased from 47 to 83% in PCa.

POSTN has been reported to be upregulated in aggressive PCa^48^, but significant changes in glycosylation have not been reported yet. Our proteomics results reaffirmed, that POSTN is overexpressed in PCa, and we also detected significant changes in both fucosylation and sialylation on POSTN N599, an increase from 24 to 72% and a decrease from 83 to 44% respectively, highlighting its’ possible importance.

Prostate tissue is known to be a rich reservoir of Prothrombin^49^, the precursor of Thrombin, which has been reported to promote prostate tumor growth, increase tumor cell seeding, and stimulate angiogenesis^50,51^. We have found that the sialylation of THRB N121 was downregulated significantly in PCa, moreover, with the largest relative difference.

Alterations of serum IgG glycosylation has been reported in many diseases, including PCa^52^, and IgG1 has been suggested as a potential target for PCa treatment^53^. We found that both IGG1 N299 and IGHG2 N176 show decreased overall galactosylation by 6.3% and 8.7% respectively. This is in line with previous studies, where one of the major differences reported was the decrease of terminal galactosylation in PCa compared to either healthy or benign prostatic disease patients^54^. Our data also shows reduced sialylation on both IGG1 N299 and IGHG2 N176 by 2.1% (corresponding to a relative change of 21.3% and 26.8% respectively), which is also in agreement with literature as reduced sialylation has been described as a major alteration in PCa compared to healthy individuals^55^.

Another glycoprotein with significant site-specific glycosylation changes was MFAP4, which has been reported to be involved in several cancers and may function as a tumor suppressor in PCa^56^. MFAP4 has been documented to have altered glycosylation in pancreatic adenocarcinoma^57^, however, not in PCa. Our results revealed that both sites of MFAP4 showed modified glycosylation in PCa: decreased sialylation on N87 and increased expression of the glycan N4H5S1F1 on N137. The latter glycoform might be a useful indicator in detecting PCa at an early stage, as this increased expression was detected between normal and G1 samples.

Most of the glycoproteins discussed above can be found in the Human Protein Atlas^58^ (apart from IGG1 and IGHG2) and are categorized in the Pathology Atlas based on Prognostic summary and Cancer specificity. Apart from PPAP, which is a protein specific to PCa, all of them are unfavorable prognostic markers in certain types of cancer (in most cases renal cancer) which suggests that these glycoproteins are heavily involved in cancer progression. This information is summarized in Supplementary Table S8 supplemented by their Secretome annotation.

It is also important to note, that these glycoproteins have been detected in biofluids previously. All glycoproteins discussed above with the exception of POSTN have been detected in urine^59^, while POSTN has been detected in serum samples^60^ of PCa patients. This suggests their potential usefulness as a clinical marker. Whether the alterations in the glycosylation of these proteins is PCa specific or not, needs further investigation, especially in the context of their biomarker status.

In conclusion, our results indicate that alterations between PCa and Normal tissue glycosylation occur primarily on the glycosite level, while overall glycosylation may be unaffected. Furthermore, altered glycosylation does not necessarily indicate differential expression on the protein level. The glycoproteins with significant differences in glycosylation were all secreted either to blood or the ECM, and most of them are characterized as an unfavorable prognostic cancer marker by the Pathology Atlas. As altered protein glycosylation in cancer has been proven to be nonrandom, this suggests that further investigation of the glycosylation, and cancer specificity of these potential prognostic markers and identification of their exact roles is reasonable and could lead to further advancement in understanding the function of glycosylation in cancer development and PCa prognosis.

## Methods

### Materials

All chemicals used were HPLC–MS grade. Acetonitrile, Water, Acetone, Formic acid, and Ammonium-bicarbonate were purchased from Merck (Darmstadt, Germany). Trifluoroacetic acid, Dithiothreitol, and Iodoacetamide were obtained from Thermo Scientific (Waltham, MA, USA). Methanol was purchased from VWR International (Debrecen, Hungary), RapiGest surfactant was obtained from Waters (Milford, MA, USA).

### Detailed information on TMAs

Four different TMA slides were purchased from US Biomax (Derwood, MD, USA): BNS19011, PR481, PR483c, PR633. All of them contained formalin-fixed paraffin-embedded (FFPE) cores with a diameter of 1.5 mm and a thickness of 5 μm. The specification sheets are available at https://biomax.us with information about each core including age, pathological Grade, Stage, and Gleason Score. Each TMA core contains on average approximately 1 µg protein.

### On-surface digestion

First, the TMA slides were baked at 60 °C for 2 h following the supplier’s instructions to prevent tissue detachment. Next, de-paraffinization was carried out by incubating the slides in different solvents/solutions sequentially as follows: xylene for 2 × 3 min, ethanol for 2 × 5 min, 90% ethanol—10% water for 3 min, 70% ethanol 30%—Water for 3 min, 10 mM NH_4_HCO_3_ (water) for 5 min and finally water for 1 min. After dewaxing, the slides were placed in antigen retrieval buffer (20 mM Tris–HCl, pH = 9.0) for 30 min at 90 °C.

Following the preparation steps, the proteins in TMA cores were reduced using RapiGest and DTT in 1 µL of 20% glycerol for 20 min at 55 °C, then alkylated using IAA in 1 µL of 25 mM ammonium bicarbonate (ABC) puffer and 20% glycerol for 20 min at room temperature in the dark. The digestion was done in a cyclic manner, each one lasting for 40 min at 37 °C in a humidified box, 5 cycles in total. In the first two cycles, LysC-Trypsin mixture was added in a 1:25 ratio, in 1 µL 50 mM ABC and 20% glycerol. Subsequently, in the last three cycles, Trypsin was added in a 1:10 ratio, in 1 µL 50 mM ABC and 20% glycerol. After the digestion steps, the extraction of the protein digest was done by repeatedly pipetting 1 µL 10% acetic acid extraction solvent five times on the cores. Peptide extracts were then dried down, and clean-up was performed using C_18_ spin columns (Thermo Scientific) using the manufacturer’s protocol. The resulting samples were dried down and stored at -20 °C for further usage.

### Acetone precipitation

Samples were reconstituted in 15 µL 1% FA and 150 µL ice-cold acetone was added and the solution was stored at -20 °C overnight. Then the samples were centrifuged at 13,000 g for 10 min, then the supernatants were removed, dried down, and stored at -20 °C. The pellet fractions were also dried down, then resuspended in 10 µL of injection solvent and subsequently stored in the autosampler unit until analysis.

### nanoUHPLC-MS(MS) analysis

Samples were analyzed using a Maxis II QTOF instrument (Bruker Daltonik GmbH, Bremen, Germany) equipped with CaptiveSpray nanoBooster ion source coupled to a Dionex UltiMate 3000 RSLCnano system (Sunnyvale, CA, USA). Peptides were separated on an Acquity M-Class BEH130 C_18_ analytical column (1.7 μm, 75 μm × 250 mm Waters, Milford, MA) using gradient elution (isocratic hold at 4% for 11 min, then elevating B solvent content to 25% in 75 min, and to 40% in 15 min) following trapping on an Acclaim PepMap100 C_18_ (5 μm, 100 μm × 20 mm, Thermo Fisher Scientific, Waltham, MA) trap column. Solvent A consisted of water + 0.1% formic acid, Solvent B was acetonitrile + 0.1% formic acid, and the sample loading buffer was 0.1% TFA and 0.01% heptafluorobutiric acid in water.

For proteomics, DDA measurements were used. The cycle time was set at 2.5 s, with a dynamic MS/MS exclusion of the same precursor ion for 2 min, or if its intensity is at least 3 times larger than previously. Preferred charge states were set between + 2 and + 5. MS spectra were acquired at 3 Hz in the 150–2200 m/z range, while MS/MS spectra at 4 or 16 Hz depending on the intensity of the precursor. For glycoproteomics MS/MS measurements, the experimental settings were similar, except for collision energies. Mixed energy spectra were collected at 100% collision energy for 80% of the cycle time and 50% collision energy for 20% of the cycle time. For single-stage MS measurements, spectra were recorded over the mass range of 300–3000 m/z at 1 Hz. Following each run, raw data were recalibrated using the Compass DataAnalysis software 4.3 (Bruker Daltonics, Bremen, Germany).

### Data analysis

Software used: MASCOT (https://www.matrixscience.com/), MaxQuant 1.6.17 (https://maxquant.org), Perseus 1.6.5.0 (https://maxquant.org/perseus/), R 3.6.1 (https://www.r-project.org/), RStudio 1.2.5001 (https://rstudio.com/), Byonic 3.8 (https://proteinmetrics.org), GlycoPattern 4.7_b30. Exact parameters used for all the software are summarized in Supplementary Table S9.

### Proteomics

Protein quantitation was performed by MaxQuant^61^ on a focused Homo Sapiens database made from combining MASCOT^62^ search results from all MS/MS analyses. The MaxQuant output was then loaded into Perseus, where proteins found in less than 60% of each sample group were removed. Subsequently, missing values were imputed from a normal distribution with the default settings for the whole matrix (down shift of 1.8 and width of 0.3). Statistical analysis was then performed, using Two-sample tests (Student’s t-test), Multiple-sample tests (ANOVA), and post-hoc tests (Tukey’s HSD). The exact settings for the statistical tests are summarized in Supplementary Table S10. Data visualizations were done in RStudio using the ggplot2 library^63^.

### Glycoproteomics

In the glycoproteomics analysis, glycosites were identified from the LC–MS/MS analysis of pooled pellet samples using Byonic^64^ with a |LogProb| value of at least 2. The same LC–MS/MS experiments were used to identify the composition of various glycans at these glycosylation sites. GlycoPattern^33^ was then used to quantify the glycopeptides based on single-stage nanoLC-MS corresponding to the linear combinations of the glycosites and glycans previously identified. The software identified the glycopeptides according to their exact mass, retention time (RT), and isotope cluster distribution, then performed label-free quantitation.

Pre-processing and statistical analysis were then carried out using R^65^ in RStudio^66^. The data were first submitted to outlier filtering, where identifications with a RT outside of the Q_1_ (first quartile)—1.5 IQR (inter-quartile range) to Q_3_ (third quartile) + 1.5 IQR range were thrown out. Then, through sequential filtering steps, any data points with an AUC less than 1000, glycopeptides identified in less than 5 samples, and samples with less than 10 glycopeptides identified were removed. Subsequently, the data were normalized using Quotient Total Area Normalization followed by log transformation^67^. The degree of fucosylation (ratio of fucosylated versus non-fucosylated glycopeptides) and sialylation (the ratio of antennae that contain sialic acid versus antennae that does not) were then calculated for every glycosite. Statistical analysis was carried out in Perseus similarly to the proteomics dataset (exact settings are summarized in Supplementary Table S10), data visualizations were done in RStudio using the ggplot2 library^63^.

### STRING

Functional Enrichment of proteins was performed in STRING^68^ for Gene Ontology (GO) Terms and Kyoto Encyclopedia of Genes and Genomes (KEGG) Pathways. The minimum required interaction score was set to the highest confidence (0.900), for active interaction sources “Textmining” was excluded.

## Supplementary Information

Supplementary Information 1.

Supplementary Information 2.

Supplementary Information 3.

Supplementary Information 4.

Supplementary Information 5.

Supplementary Information 6.

Supplementary Information 7.

Supplementary Information 8.

Supplementary Information 9.

## Data Availability

Experimental data has been submitted to the MassIVE data repository with the ID: MSV000087329.

## References

[1] Siegel RL, Miller KD, Jemal A (2016). Cancer statistics, 2016. CA Cncer J. Clin..

[2] Mottet N (2017). EAU-ESTRO-SIOG guidelines on prostate cancer. Part 1: Screening, diagnosis, and local treatment with curative intent. Eur. Urol..

[3] Sandhu GS, Andriole GL (2012). Overdiagnosis of prostate cancer. J. Natl. Cancer Inst. Monogr..

[4] Bulten W (2020). Artificial intelligence assistance significantly improves Gleason grading of prostate biopsies by pathologists. Mod. Pathol..

[5] Corcoran NM (2012). Underestimation of Gleason score at prostate biopsy reflects sampling error in lower volume tumours. BJU Int..

[6] Viste E (2020). Effects of replacing PSA with Stockholm3 for diagnosis of clinically significant prostate cancer in a healthcare system–the Stavanger experience. Scand. J. Prim. Health Care.

[7] Wu JT, Liu GH (1998). Advantages of replacing the total PSA assay with the assay for PSA-α1-antichymotrypsin complex for the screening and management of prostate cancer. J. Clin. Lab. Anal..

[8] Brown AM (2015). Recent advances in image-guided targeted prostate biopsy. Abdom. Imaging.

[9] Ahdoot M (2020). MRI-targeted, systematic, and combined biopsy for prostate cancer diagnosis. N. Engl. J. Med..

[10] Ström P (2020). Artificial intelligence for diagnosis and grading of prostate cancer in biopsies: A population-based, diagnostic study. The Lancet Oncol..

[11] Bulten W (2020). Automated deep-learning system for Gleason grading of prostate cancer using biopsies: A diagnostic study. The Lancet Oncol..

[12] Heitzer E, Haque IS, Roberts CE, Speicher MR (2019). Current and future perspectives of liquid biopsies in genomics-driven oncology. Nat. Rev. Genet..

[13] Erickson A (2018). New prostate cancer grade grouping system predicts survival after radical prostatectomy. Hum. Pathol..

[14] Epstein JI (2018). Prostate cancer grading: A decade after the 2005 modified system. Mod. Pathol..

[15] Srigley JR (2019). Controversial issues in Gleason and International Society of Urological Pathology (ISUP) prostate cancer grading: proposed recommendations for international implementation. Pathology.

[16] Abeshouse A (2015). The molecular taxonomy of primary prostate cancer. Cell.

[17] Neuhaus J (2021). Special issue “Diagnostic biomarkers in prostate cancer”. Diagnostics.

[18] Shukla S (2020). Oxidative stress and antioxidant status in high-risk prostate cancer subjects. Diagnostics.

[19] Saxby H, Mikropoulos C, Boussios S (2020). An update on the prognostic and predictive serum biomarkers in metastatic prostate cancer. Diagnostics.

[20] Jakobsen NA, Hamdy FC, Bryant RJ (2016). Novel biomarkers for the detection of prostate cancer. J. Clin. Urol..

[21] Filella X, Fernández-Galan E, Bonifacio RF, Foj L (2018). Emerging biomarkers in the diagnosis of prostate cancer. Pharmacogenom. Pers. Med..

[22] Scott E, Munkley J (2019). Glycans as biomarkers in prostate cancer. Int. J. Mol. Sci..

[23] Matsumoto T (2019). Serum N-glycan profiling is a potential biomarker for castration-resistant prostate cancer. Sci. Rep..

[24] Tkac J (2019). Prostate-specific antigen glycoprofiling as diagnostic and prognostic biomarker of prostate cancer. Interface Focus.

[25] Schwamborn K (2007). Identifying prostate carcinoma by MALDI-Imaging. Int. J. Mol. Med..

[26] Srinivas PR, Srivastava S, Hanash S, Wright GL (2001). Proteomics in early detection of cancer. Clin. Chem..

[27] Schiess R, Wollscheid B, Aebersold R (2009). Targeted proteomic strategy for clinical biomarker discovery. Mol. Oncol..

[28] Sardana G, Jung K, Stephan C, Diamandis EP (2008). Proteomic analysis of conditioned media from the PC3, LNCaP, and 22Rv1 prostate cancer cell lines: discovery and validation of candidate prostate cancer biomarkers. J. Proteome Res..

[29] Cao W (2021). Recent advances in software tools for more generic and precise intact glycopeptide analysis. Mol. Cell. Proteom..

[30] Turiák L (2019). High sensitivity proteomics of prostate cancer tissue microarrays to discriminate between healthy and cancerous tissue. J. Proteom..

[31] Turiák L (2014). Workflow for combined proteomics and glycomics profiling from histological tissues. Anal. Chem..

[32] Turiák L (2019). Site-specific N-glycosylation of HeLa cell glycoproteins. Sci. Rep..

[33] Ozohanics O, Turiák L, Puerta A, Vékey K, Drahos L (2012). High-performance liquid chromatography coupled to mass spectrometry methodology for analyzing site-specific N-glycosylation patterns. J. Chromatogr. A.

[34] Christiansen MN (2014). Cell surface protein glycosylation in cancer. Proteomics.

[35] Figel S, Gelman I (2011). Focal adhesion kinase controls prostate cancer progression via intrinsic kinase and scaffolding functions. Anti-Cancer Agents Med. Chem..

[36] Wang Y (2016). Smooth muscle contraction and growth of stromal cells in the human prostate are both inhibited by the Src family kinase inhibitors, AZM475271 and PP2. Br. J. Pharmacol..

[37] Wang X, Li S (2014). Protein mislocalization: Mechanisms, functions and clinical applications in cancer. Biochim. Biophys. Acta Rev. Cancer.

[38] Liang P, Pardee AB (2003). Analysing differential gene expression in cancer. Nat. Rev. Cancer.

[39] Wang M (2017). Role of tumor microenvironment in tumorigenesis. J. Cancer.

[40] Varki, A. *et al. Essentials of Glycobiology* [internet]. Chapter 9. (2015).

[41] Pinho SS, Reis CA (2015). Glycosylation in cancer: mechanisms and clinical implications. Nat. Rev. Cancer.

[42] Kawahara R (2021). The complexity and dynamics of the tissue glycoproteome associated with prostate cancer progression. Mol. Cell. Proteom..

[43] Drake RR (2017). MALDI mass spectrometry imaging of N-linked glycans in cancer tissues. Adv. Cancer Res..

[44] Zhang C (2019). Elevated serum sialic acid levels predict prostate cancer as well as bone metastases. J. Cancer.

[45] Zhu Y-P (2015). Reactive stroma component COL6A1 is upregulated in castration-resistant prostate cancer and promotes tumor growth. Oncotarget.

[46] Shah P (2015). Integrated proteomic and glycoproteomic analyses of prostate cancer cells reveal glycoprotein alteration in protein abundance and glycosylation. Mol. Cell. Proteom..

[47] Muniyan S (2013). Human prostatic acid phosphatase: Structure, function and regulation. Int. J. Mol. Sci..

[48] Tian Y, Bova GS, Zhang H (2011). Quantitative glycoproteomic analysis of optimal cutting temperature-embedded frozen tissues identifying glycoproteins associated with aggressive prostate cancer. Anal. Chem..

[49] Kohli M (2011). Thrombin expression in prostate: A novel finding. Cancer Inv..

[50] Adams G (2015). Thrombin and factor XII drive prostate tumor growth in vivo. Blood.

[51] Nierodzik ML, Karpatkin S (2006). Thrombin induces tumor growth, metastasis, and angiogenesis: Evidence for a thrombin-regulated dormant tumor phenotype. Cancer Cell.

[52] Gudelj I, Lauc G, Pezer M (2018). Immunoglobulin G glycosylation in aging and diseases. Cell. Immunol..

[53] Xu Y (2016). IgG silencing induces apoptosis and suppresses proliferation, migration and invasion in LNCaP prostate cancer cells. Cell. Mol. Biol. Lett..

[54] Kazuno S (2016). Glycosylation status of serum immunoglobulin G in patients with prostate diseases. Cancer Med..

[55] Papakonstantinou M (2019). N-Glycosylation of IgG Immunoglobulin and its clinical significance. J. Biomed..

[56] Yang J (2019). Integrated analysis of microfibrillar-associated proteins reveals MFAP4 as a novel biomarker in human cancers. Epigenomics.

[57] Guerrero PE (2021). Microfibril associated protein 4 (MFAP4) is a carrier of the tumor associated carbohydrate sialyl-Lewis x (sLex) in pancreatic adenocarcinoma. J. Proteom..

[58] Uhlen M (2017). A pathology atlas of the human cancer transcriptome. Science.

[59] Dong M (2020). Urinary glycoproteins associated with aggressive prostate cancer. Theranostics.

[60] Gabriele C (2019). High-throughput detection of low abundance sialylated glycoproteins in human serum by TiO 2 enrichment and targeted LC-MS/MS analysis: Application to a prostate cancer sample set. Anal. Bioanal. Chem..

[61] Cox J, Mann M (2008). MaxQuant enables high peptide identification rates, individualized ppb-range mass accuracies and proteome-wide protein quantification. Nat. Biotechnol..

[62] Brosch M, Yu L, Hubbard T, Choudhary J (2009). Accurate and sensitive peptide identification with Mascot Percolator. J. Proteome Res..

[63] Hadley W (2016). Ggplot2: Elegrant Graphics for Data Analysis.

[64] Bern M, Kil YJ, Becker C (2012). Byonic: Advanced peptide and protein identification software. Curr. Protoc. Bioinform..

[65] R. Team. *R: A Language and Environment for Statistical Computing*. (2013).

[66] RStudio Team. *RStudio: Integrated Development Environment for R*. (PBC, 2020).

[67] Benedetti E (2020). Systematic evaluation of normalization methods for glycomics data based on performance of network inference. Metabolites.

[68] Szklarczyk D (2019). STRING v11: Protein–protein association networks with increased coverage, supporting functional discovery in genome-wide experimental datasets. Nucleic Acids. Res..

