# Supplementary material for: Alterations in protein expression and site-specific N-glycosylation of prostate cancer tissues
Source: Sci Rep. 2021 Aug 5;11:15886. doi: 10.1038/s41598-021-95417-5 (PMC8342536; doi:10.1038/s41598-021-95417-5)
Supplement: Supplementary file 1 — Supplementary Information 1. [file 41598_2021_95417_MOESM1_ESM.pdf]

Supplementary data for

Alterations in protein expression and site-specific *N*-glycosylation of prostate cancer tissues

Table of contents:

1.: Supplementary Table S1

2.: Supplementary Table S8

3.: Supplementary Figure S1

4.: Supplementary Figure S2

5.: Supplementary Figure S3

6.: Supplementary Figure S4

7.: Supplementary Figure S5

8.: Supplementary Figure S6

9.: Supplementary Figure S7

10.: Supplementary Figure S8

Supplementary Tables S2-S7 and S9-S10 are provided separately as text files:

Supplementary Table S2: Over- and underexpressed proteins between healthy and cancerous prostate tissues.

Supplementary Table S3: Proteins with significant changes among various prostate cancer grades and healthy tissues (quantitative data is provided as Z-scores).

Supplementary Table S4: GOBP terms identified by STRING in proteins underexpressed in prostate cancer.

Supplementary Table S5: KEGG terms identified by STRING in proteins underexpressed in prostate cancer.

Supplementary Table S6: GOBP terms identified by STRING in proteins overexpressed in prostate cancer.

Supplementary Table S7: KEGG terms identified by STRING in proteins overexpressed in prostate cancer.

Supplementary Table S9: Parameters for the softwares used: MASCOT, MaxQuant, Byonic and GlycoPattern.

Supplementary Table S10: Parameters for the different statistical tests used.

Supplementary Table S1. The terms used to characterize N-glycosylation and their short explanation

Term	Explanation
Glycan type	The proportion of glycans classified as either Complex, Oligomannose, Hybrid or Paucimannose.
Branching	The proportion of glycans with a set number of antennae versus all of them - for Complex type glycans only.
Sialylation	The proportion of antennae that contain a Sialic acid residue versus all complete antennae.
Fucosylation	The proportion of glycans with a Fucose residue versus all Complex, Hybrid and Paucimannose glycans.
Galactosylation	The proportion of complete antennae versus all antennae.

Supplementary Table S8. Summary of multiple classifications from the Human Protein Atlas for the glycoproteins discussed in the paper

Protein	Prognostic summary	Cancer specificity	Secretome annotation
CO6A2	Unfavorable in Renal cancer	Low	Extracellular matrix
POSTN	Unfavorable in Renal, Lung, Stomach cancer	Breast cancer	Extracellular matrix
THRB	Unfavorable in Renal cancer	Low	Blood
PPAP	-	Prostate cancer	Blood
A1AG1	Unfavorable in Renal cancer	Liver cancer	Blood
MFAP4	Unfavorable in Renal, Ovarian, Urothelial cancer, Favorable in Neck and Head cancer	Low	Extracellular matrix
PGS1	Unfavorable in Renal cancer	Low	Extracellular matrix

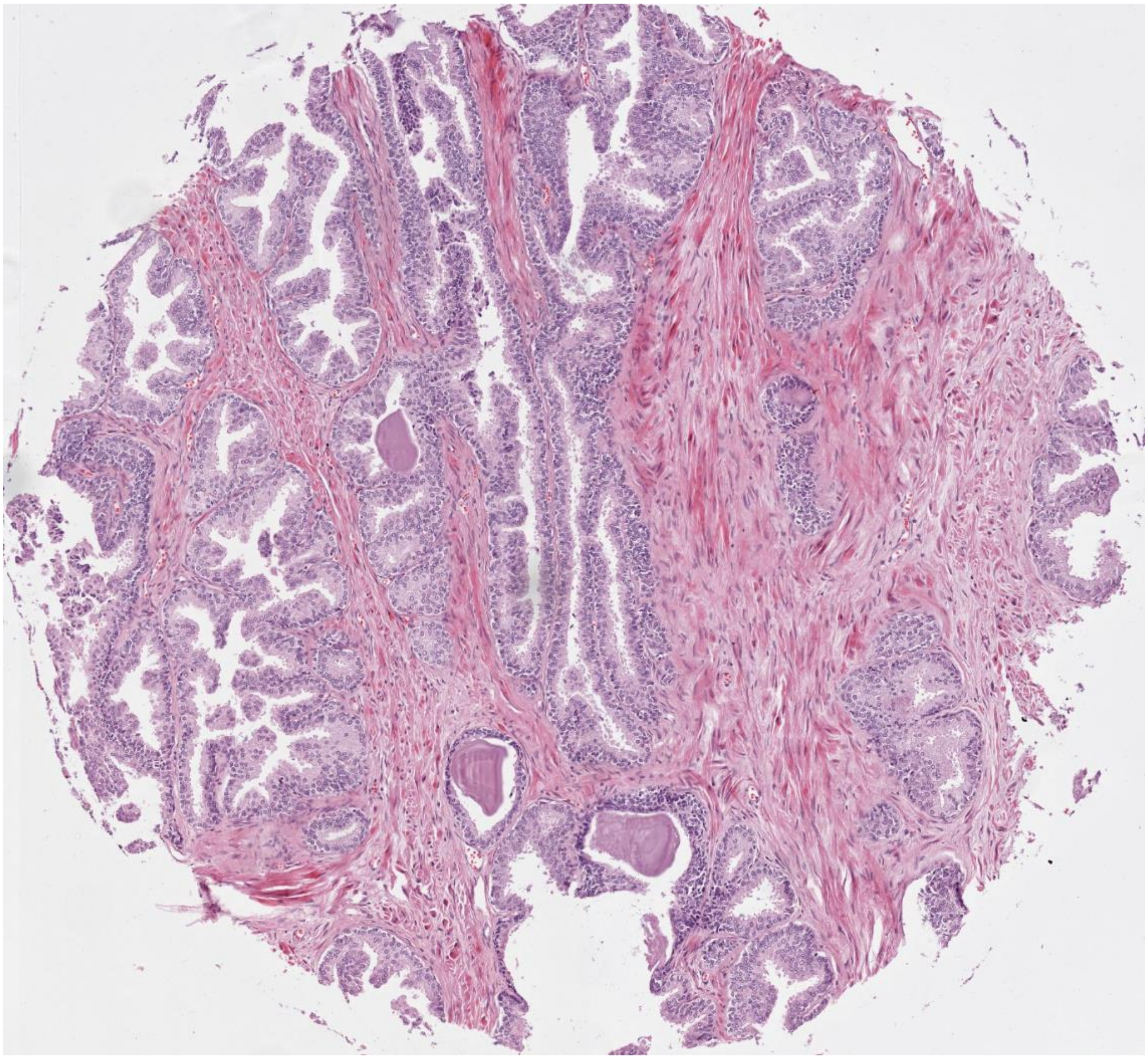

Supplementary Figure S1: An example of a stained healthy prostate TMA sample. Original image from US Biomax webpage (https://www.biomax.us/tissue-arrays/Prostate/PR633).

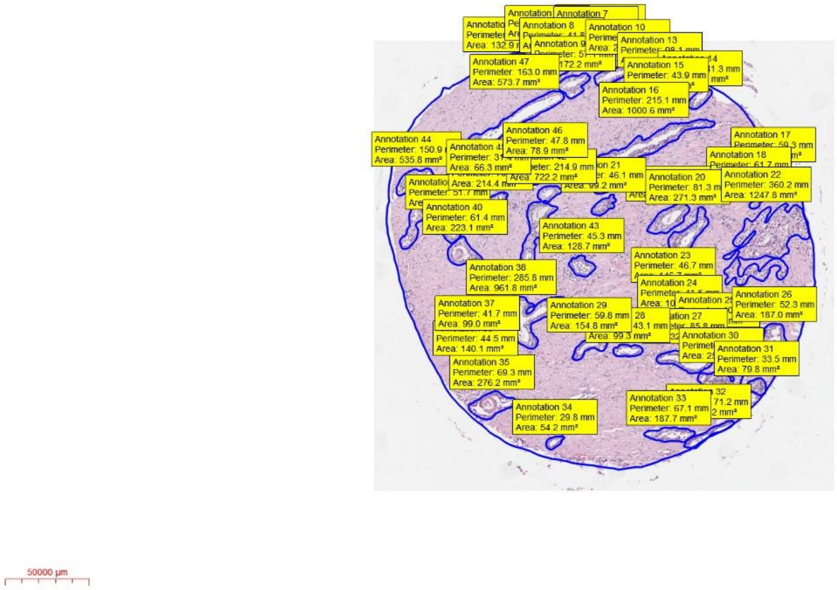

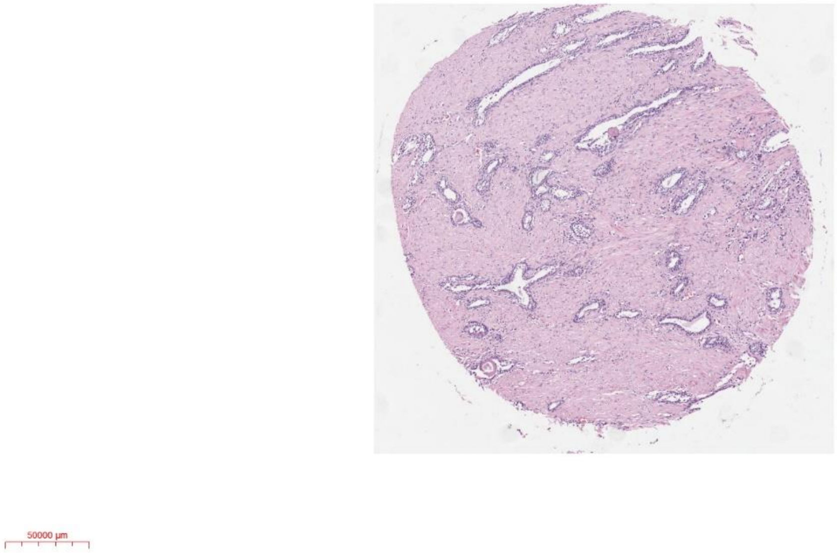

Supplementary Figure S2: An example of a stained Grade 1 TMA sample, in original, and in pathologically annotated form. Original image from US Biomax webpage (https://www.biomax.us/tissue-arrays/Prostate/PR633). The manual annotation of cancerous and non-cancerous tissue areas was carried out using CaseViewer software version 2.4.0.119028 (3DHistech Ltd., Budapest, Hungary).

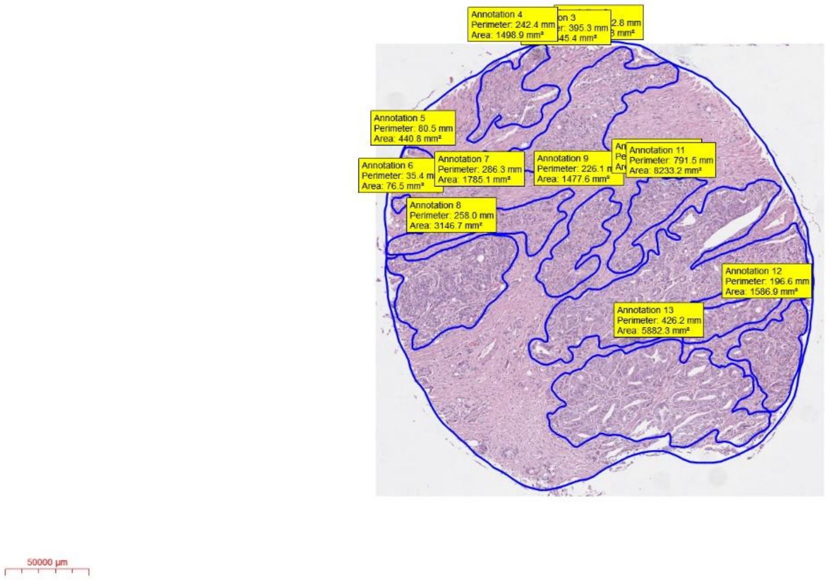

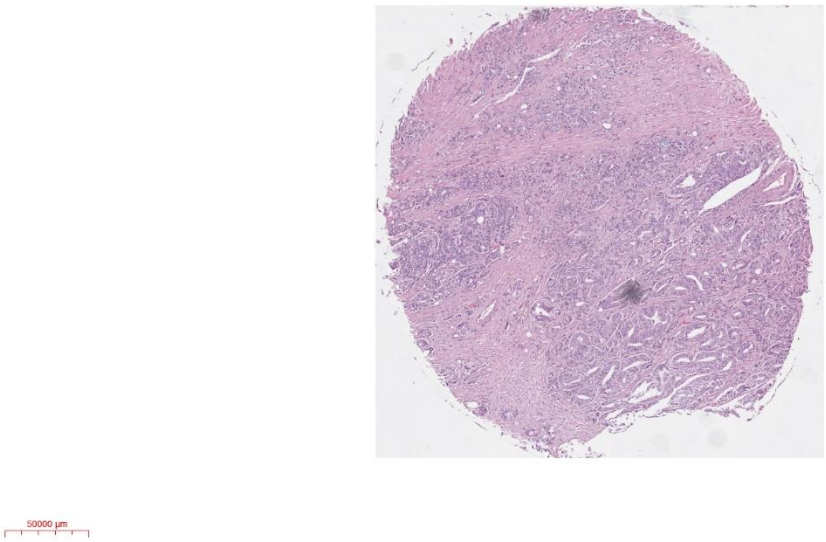

Supplementary Figure S3: An example of a stained Grade 2 TMA sample, in original, and in pathologically annotated form. Original image from US Biomax webpage (https://www.biomax.us/tissue-arrays/Prostate/PR633). The manual annotation of cancerous and non-cancerous tissue areas was carried out using CaseViewer software version 2.4.0.119028 (3DHistech Ltd., Budapest, Hungary).

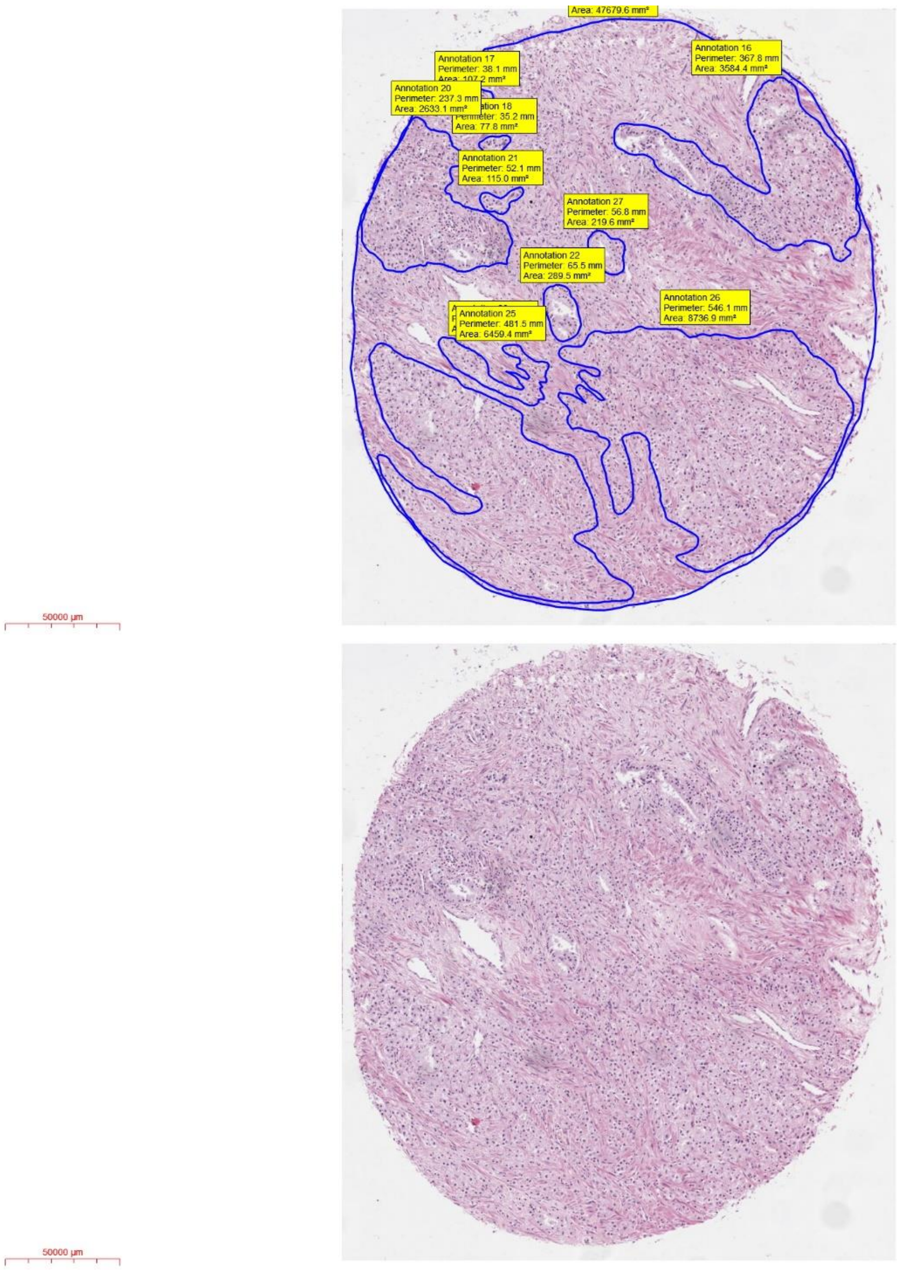

Supplementary Figure S4: An example of a stained Grade 3 TMA sample, in original, and in pathologically annotated form. Original image from US Biomax webpage (https://www.biomax.us/tissue-arrays/Prostate/PR633). The manual annotation of cancerous and non-cancerous tissue areas was carried out using CaseViewer software version 2.4.0.119028 (3DHistech Ltd., Budapest, Hungary).

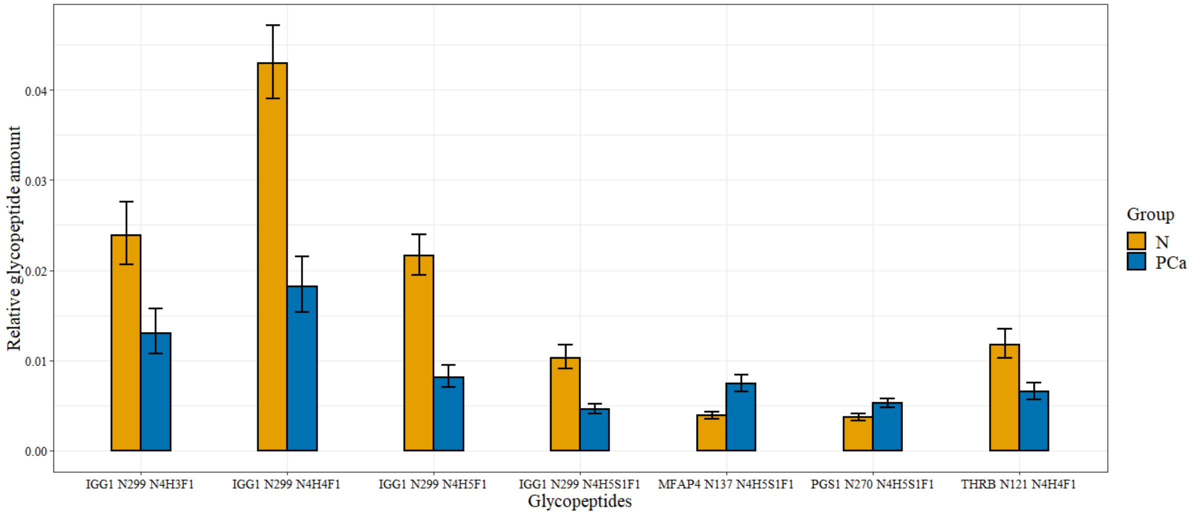

Supplementary Figure S5: Glycopeptides with significantly different abundances between healthy and cancerous tissues with standard error.

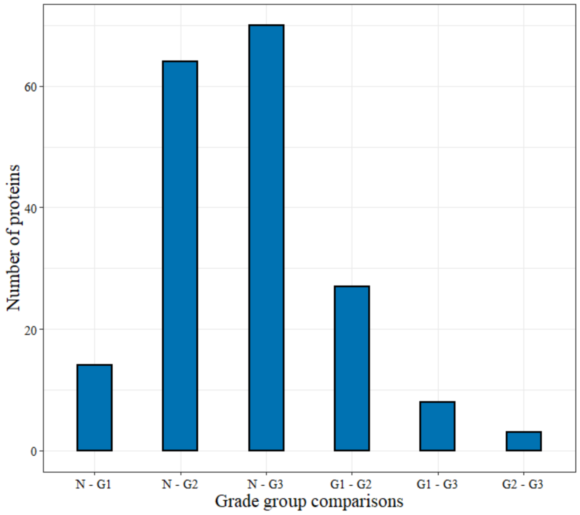

Supplementary Figure S6: The number of proteins with significant differences in the different pairwise comparisons between prostate cancer grades and healthy tissue.

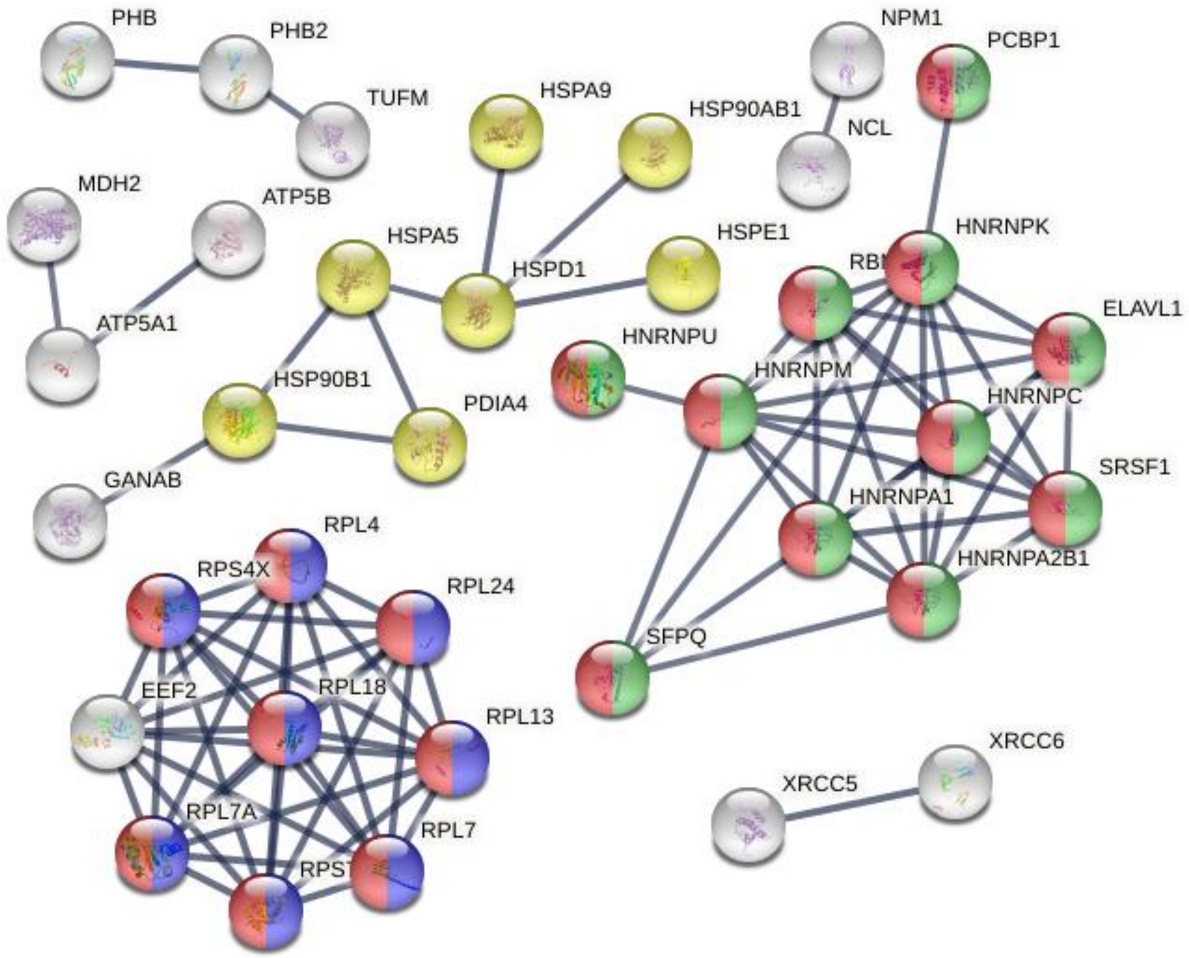

Supplementary Figure S7: PPI Networks constructed in STRING for proteins upregulated in prostate cancer.

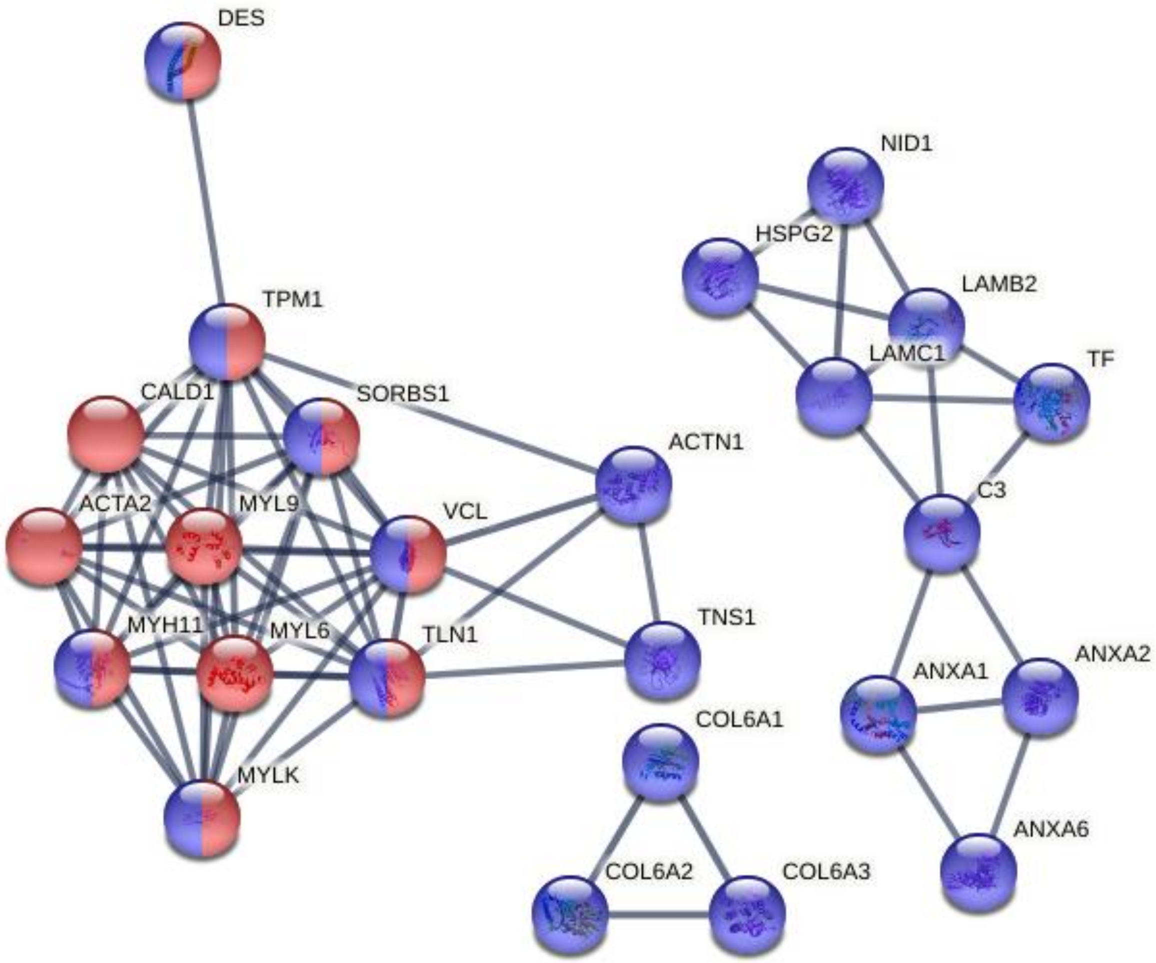

Supplementary Figure S8: PPI Networks constructed in STRING for proteins downregulated in prostate cancer.
